# The efficacy and safety of esketamine in the treatment of major depressive disorder with suicidal ideation: study protocol for a randomized controlled trial

**DOI:** 10.1186/s12888-022-04388-y

**Published:** 2022-11-30

**Authors:** Haiyan Liu, Xiaofeng Lan, Chengyu Wang, Fan Zhang, Ling Fu, Weicheng Li, Yanxiang Ye, Zhibo Hu, Ziyuan Chao, Yuping Ning, Yanling Zhou

**Affiliations:** 1 Guangdong Engineering Technology Research Center for Translational Medicine of Metal Disorders, Guangzhou, China; 2 The Affiliated Brain Hospital of Guangzhou Medical University (Guangzhou Huiai Hospital), Guangzhou, China; 3 The First School of Clinical Medicine, Southern Medical University, Guangzhou, China

**Keywords:** Clinical trial, Esketamine, Major depressive disorder, Suicide

## Abstract

**Background:**

Major depressive disorder (MDD) is a high risk factor for suicide, with up to 20% of MDD patients attempting suicide during their lifetime. Current treatments for MDD are slow onset of action, low efficiency, and the inability to control suicidal behaviors quickly and effectively. Intravenous ketamine has been shown to have a rapid but transient antidepressant effect, but there is still lack evidence on the efficacy and safety of intravenous esketamine in reducing suicidal ideation and depressive symptoms in MDD patients with suicidal ideation. We designed a study to investigate the effect of short-term repeated intravenous infusion of esketamine three times in MDD patients with suicidal ideation.

**Methods:**

This study features a randomized, double-blind, placebo-controlled trial (RCT) comparing short-term repeated intravenous infusions of esketamine with placebo as a supplement to conventional antidepressants with an intervention period of 6 days and one infusion every other day, followed by 4 weeks of follow-up. These methods support the examination of the efficacy, safety, tolerability, and mechanism of action of short-term repeated intravenous infusions of esketamine in MDD patients with suicidal ideation.

**Discussion:**

This is the first RCT to explore the efficacy and safety of short-term repeated infusion of esketamine on suicidal ideation and depressive symptoms in MDD patients with suicidal ideation. If proven effective and tolerated, it will provide evidence for rapid and effective treatment of suicidal ideation and depressive symptoms in MDD individuals with suicidal ideation.

**Trial registration:**

Chinese Clinical Trial Register, ChiCTR2000041232. Registered 22 December 2020.

## Background

Major depressive disorder (MDD), as a major contributor to the global burden of disease, is a common mental illness with high lifetime prevalence and disability rates [1, 2]. Suicide is the most serious public health crisis, being the second leading cause of death among 15–29 years worldwide [3]. Evidence suggests that MDD is a high risk factor for suicide, with approximately 2/3 of MDD patients experiencing suicidal ideation and up to 20% of MDD patients attempting suicide during their lifetime [2, 4]. Despite this, current conventional antidepressant treatment faces great dilemmas: slow onset of action, low efficiency, and the inability to control risky behavioral suicides quickly and effectively [5, 6]. Therefore, it is particularly important to explore drugs that rapidly improve depressive symptoms, especially suicidal ideation/behavior, in MDD patients.

Ketamine, a non-competitive N-methyl-D-aspartate (NMDA) receptor antagonist, has been shown to have rapid and potent as well as relatively long-lasting antidepressant efficacy in animal and clinical studies [7–9]. Of note, the U.S. Food and Drug Administration (FDA) officially approved a nasal spray form of esketamine (the S-enantiomer of ketamine) for the treatment of refractory depression (TRD) in 2019, the same year the drug was approved for the same indication in Europe.

An increasing number of studies in recent years have focused on ketamine in the treatment of suicide. Several studies have shown rapid efficacy of multiple ketamine infusions for the alleviation of suicidal ideation [10, 11]. A Meta-analysis showed that a single dose of intravenous ketamine provides rapid alleviation of suicidal ideation, with its antisuicidal effect manifesting within 24 hours [11]. This rapid antisuicidal effect is well suited to clinical needs. Also, this review also shown that the antisuicidal effect of ketamine is remained significant after adjusting for concurrent changes in severity of depressive symptoms. Our team conducted a clinical study on this in the early stage. We used six-dose infusions of ketamine (0.5 mg/kg) over 2 weeks to treat a total of 86 depressed patients with suicidal ideation. 57% of them had immediate remission of suicidal symptoms after the first dose, and the remission rate reached 65.1% at the end of six infusions, indicating that ketamine has a rapid and strong efficacy in alleviating suicidal ideation [12]. On August 3, 2020 the FDA also approved its enantiomer esketamine nasal spray in combination with oral antidepressants for the treatment of depressive symptoms in adults with depression with suicidal ideation/behavior. However, the literature on repeated infusion of esketamine for the treatment of suicidal ideation in MDD patients is sparse and mostly open-label, and controlled trials, particularly in adolescents, remain blank, requiring larger controlled studies.

In addition, our previous study showed that nearly 40% of MDD patients failed to response to ketamine, consistent with other studies [8, 13]. Therefore, finding markers for predicting treatment response and selecting the best treatment for each individual is a key issue. Inflammatory factors as well as neurotrophic factors are popular among alternative response biomarkers. Evidence showed that antidepressant therapy is ineffective in depressed patients with increased inflammatory levels [14, 15]; and neurotrophic factors levels increased after antidepressant treatment [16, 17]. However, current results are inconsistent [18, 19]. Additional studies showed abnormalities in brain structure and function in MDD patients [20, 21], and their brain structure and function change after treatment [22, 23]. The above mentioned biomarkers would be very valuable information in ketamine’s clinical study.

### Objectives

In summary, treatment of suicidal ideation in MDD patients with suicidal ideation is currently very challenging and few effective rapid interventions have been found in robust trials of sufficient quality. So far, intravenous ketamine has been preliminarily found to have a rapid but transient anti-suicide effect, but there is still lack evidence on the efficacy and safety of repeated intravenous esketamine in reducing suicidal ideation and depressive symptoms in MDD patients with suicidal ideation. Therefore, this randomized, double-blind, placebo-controlled trial was designed to evaluate the effect of esketamine for suicidal ideation in MDD patients with suicidal ideation by comparing esketamine plus conventional therapy and placebo plus conventional therapy, as an additional strategy in addition to conventional therapy. At the same time, the effects of esketamine on self-reported severity of depression, cognitive function, quality of life, safety and tolerance were investigated. In addition, we also aim to determine biological markers for predicting esketamine’s treatment response and explore the mechanisms associated with its antidepressant and antisuicidal effects.

Esketamine and placebo both combined with conventional therapy are conditions we wish to evaluate with difference in suicidal ideation as the primary outcome measure. We hypothesized that intervention with esketamine in combination with conventional therapy would reduce suicidal ideation better than placebo in combination with conventional therapy in MDD patients with suicidal ideation.

## Methods/ design

### Study design and setting

The trial is a prospective, randomized, double-blind, interventional, controlled study with 2 parallel arms as a supplement to conventional antidepressant medications: one group was esketamine (intervention) and the other was placebo (control). Our study control used midazolam, which has no established antidepressant or antisuicidal effects and is generally considered to be the best effective control for ketamine. Throughout the study, there was no interference or restriction for the patient ‘s physician in charge to adjust psychiatric medications according to the patient’ s condition, but phenobarbital could not be used throughout the acute treatment period, while the patients were not allowed to undergo concurrent electroconvulsive therapy (ECT) and repetitive transcranial magnetic stimulation (rTMS).

This study was approved by the Ethics Committee of the Affiliated Brain Hospital of Guangzhou Medical University (Ethics number: 2020 [057]) and was currently registered with the China Clinical Trials Center under the registration number ChiCTR2000041232. The study was supervised by the Hospital Department of Education and Information (HDEI) and the Academic Management Committee of the Affiliated Brain Hospital of Guangzhou Medical University. HDEI staff audit the trial twice a year and provide feedback to the investigators.

This study will be conducted at the Affiliated Brain Hospital of Guangzhou Medical University. The institution conducting the study procedures will provide the necessary hospital structure, medications, and human resources.

### Recruitment

Our research will be advertised on the hospital’s online public website and other media platforms. Prior to screening, all potential participants will receive a verbal and written explanation of the study process, potential benefits and risks. They will all be informed that participation is voluntary and that they can withdraw at any time for any reason. All subjects and their legal guardians must give full informed consent to this project and sign a written informed consent form before being enrolled in this study.

### Patients

The study will enroll in patients with moderate to severe depression, and patients will be selected for the study based on the inclusion and exclusion criteria listed in Table 1. During the study period, if a patient has a medical emergency or a change in the patient’s condition, the investigator may decide whether the patient will withdraw from the study.

**Table 1:** Inclusion and exclusion criteria

Subject eligibility	
Inclusion criteria	1. Aged 13–65 years male and female inpatients;
	2. Met the criteria for MDD according to the DSM-5 without psychotic features;
	3. Measured by the 17-item Hamilton Depression Rating Scale (HAMD-17) total score ≥ 17;
	4. Patients with current suicidal ideation for ≥3 months as measured by a clinician-rated Columbia suicide severity rating scale (C-SSRS) ideation score ≥ 1 and a self-report Beck Scale for Suicide Ideation (SSI) item 4 or 5 score ≥ 2;
	5. Subjects with primary school education or above;
	6. The subjects who understand the content of the study are willing to join the study and sign the written informed consent.
Exclusion criteria	1. Patients with other mental disorders diagnosed by DSM-5, including schizophrenia, bipolar disorder, substance related disorder and addiction disorder (except anxiety disorder);
	2. Patients with psychotic symptoms in recent 3 months who scored more than 1 in any of the positive symptom items of BPRS (hallucination, exaggeration, suspicion and unusual thinking content);
	3. Patients with high suicide risk: had suicidal behavior (defined as self-injuring behavior with intention to die) in the past six months;
	4. Patients with history of nervous system diseases such as epilepsy and dementia, or patients with clinically significant and uncontrolled cardiovascular, kidney, liver, gastrointestinal, blood, immune, endocrine, pulmonary diseases or narrow angle glaucoma (judged according to history, physical examination or auxiliary examination);
	5. Patients with allergic reaction to ketamine;
	6. Patients with positive urine toxicology screening at screening or baseline;
	7. Subjects with positive serum / urine pregnancy test or breast-feeding at the time of screening, or planned to be pregnant during the study.

Abbreviations: *MDD*: major depressive disorder; *DSM-5*: Diagnostic and Statistical Manual of Mental Disorders

### Randomization and blinding

The raters and participants as well as their clinician will be kept blind to the assigned treatment at randomization. A designated psychiatrist, who is not involved in subjects registration or assessment scales, will generate the random assignment sequence through the SPSS random number generator program and the two drugs will be randomly assigned to subjects for treatment in a 1:1 ratio. If unblinding is required at the end of follow-up or during the course of the study for reasons such as serious side effects, the psychiatrist esponsible for randomly generated assignment sequences will also perform unblinding according to the randomized sequence number. The psychiatrist will prepare the study medications with a nurse who is not involved in other aspects of the study in each participating department. Since both drugs will be diluted in saline bottles, they cannot be identified by the naked eye. Blinding will occur at the level of the clinician and the assessor, the participant. The only professional with knowledge of the drugs infused is the investigator in charge of the distribution process, who is not involved in any clinical evaluation.

### Intervention

**Fig. 1:**
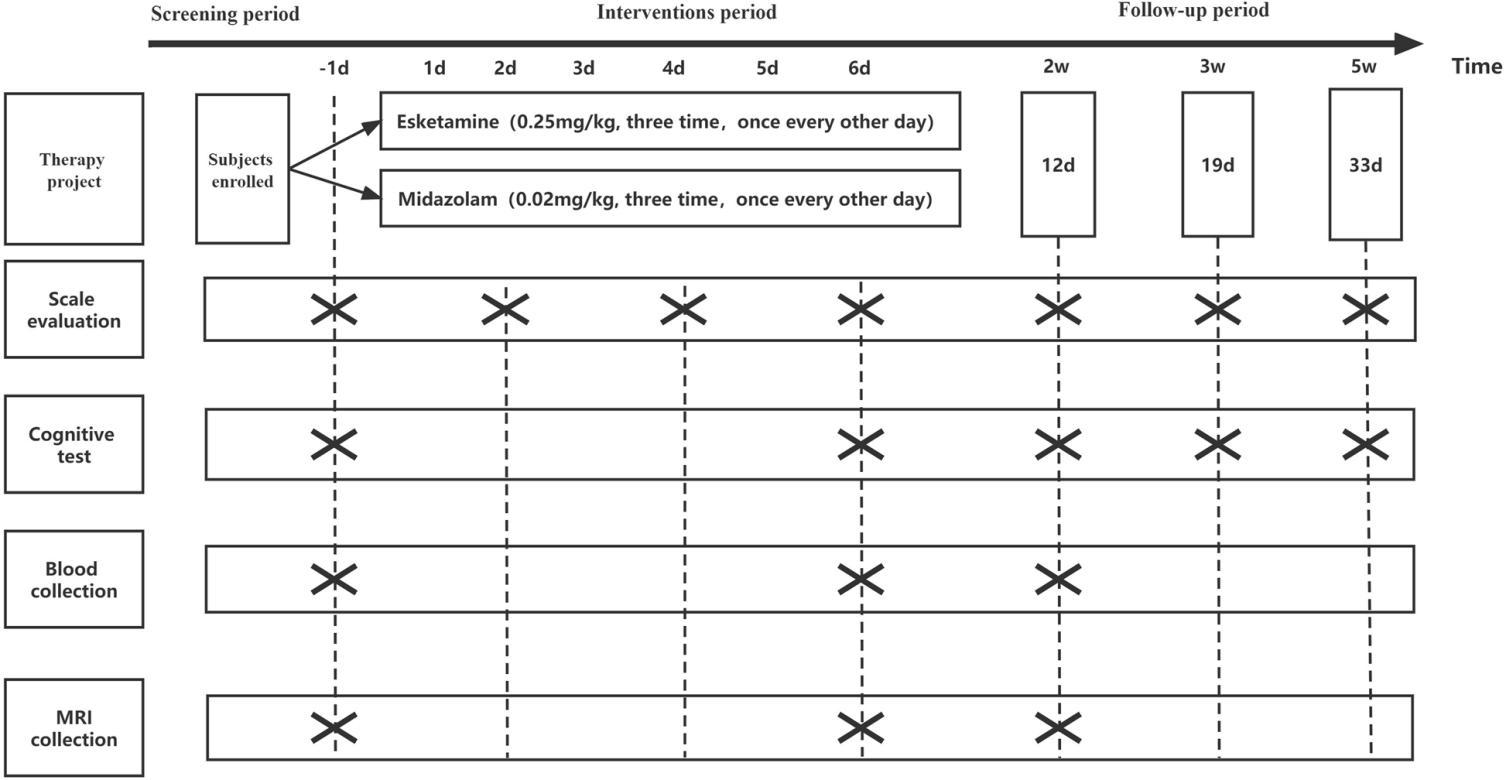
Research flow chart

All patients who meet the inclusion criteria will enter into this study and will be treated with one of two randomized drug infusion in addition to oral antidepressant medication, receiving either 0.25 mg/kg of esketamine (Jiangsu Hengrui Medicine Co.,Ltd. Specifications:2 ml:50 mg National Drug Approval:H20193336) or 0.02 mg/kg of midazolam (Commercial brand: Jiangsu Nhwa Pharmaceutical Co.,Ltd.， Specifications:2 ml: 10 mg，National Drug Approval:H10980025) added to 50 ml of 0.9% sodium chloride infusion, pumped once every other day for three times over 40 minutes. Infusions will be administered in the ward in the morning and a psychiatrist is involved during each infusion. Fig. 1 represents the research procedure schematically.

### Safety and monitoring

All participants fasted overnight (at least 8 hours) prior to each infusion until 30 minutes after the infusion. Blood pressure and heart rate will be recorded at 10-minute intervals during each infusion. After the infusion is completed, participants are instructed to rest in a hospital bed under medical supervision for at least 30 minutes until free of discomfort while blood pressure and heart rate is checked again. Patients will also be asked to report any other possible adverse events not only during the intervention but also at 30 minutes, 24 hours after the infusion and at the follow-up phases (Days 12, 19 and 33). The investigator will decide to withdraw the patient from the study if the patient encounter the following withdrawal criteria.

Withdrawal Criteria:

Patients have the right to withdraw from the study at any time for any reason.

In addition, patients were excluded from the study if they answered “yes” to the following statements.

(i) Somatic contraindications, including the following: a. Unstable vital signs: systolic or diastolic blood pressure increased by 20% from baseline, or systolic blood pressure > 180 mmHg and diastolic blood pressure > 100 mmHg, or heart rate > 110 beats/min, or heart rate < 40 beats/min, or respiration > 30 breaths/min, or respiration < 10 breaths/min; b. Excessive sedation and unresponsiveness to verbal commands; c. Partial or complete airway obstruction; d. Blood oxygen saturation (SpO2) < 95% for more than 5 minutes.
(ii) Intolerable adverse reactions.
(iii) Manic episode or severe psychotic symptoms.
(iv) Deteriorating clinical condition and risk assessment indicating unsuitability for continued participation in this trial (e.g., recurrent severe suicidal tendencies to self-injury), etc.

Patients who withdraw from treatment early are encouraged to continue to schedule regular outpatient visits to complete the study safety and efficacy assessments as if they were still on the study medication. If a patient discontinues study drug therapy, he or she immediately enters the follow-up phase.

### Efficacy evaluations/endpoints

The primary outcome is the change in suicidal ideation from Day 0 to Day 6 as assessed by clinician-rated Columbia suicide severity rating scale (C-SSRS) [24] Ideation and Intensity during the double-blind phase. The clinician-rated C-SSRS quantifies suicidal ideation and behavior through a semistructured interview. The first 5 items of the scale refer to suicidal ideation and have binary yes/no responses (yes = 1, no = 0): wish to be dead, nonspecific active suicidal thoughts, suicidal thoughts with methods, suicidal intent, and suicidal intent with plan. The next 5 items address the intensity of suicidal ideation: frequency, duration, controllability and deterrents of ideation, and reasons for ideation, and each item scales 0 (suicidal ideation denied) to 5 (suicidal ideation with a plan, i.e. severe suicidal ideation) to 5 (planned suicidal ideation, i.e., severe suicidal ideation), with a total score range of 0 to 25. C-SSRS assessment will be performed at baseline (day 0, past week recall), then 24 hours after each infusion (Days 2, 4, and 6, modified from past 24-hour recall), and then again 1, 2, and 4 weeks after the final infusion (Days 12, 19, and 33, based on past week recall).

Key secondary outcomes include changes in depressive symptoms assessed by the clinician-rated Montgomery-Osberg Depression Rating Scale (MADRS) [25] from day 0 to day 6 and given at the same time points and same recall periods as the C-SSRS.

The MADRS will be used to measure depressive symptoms because it is more sensitive in adult to ketamine-related acute changes (e.g., hours or less) than the HAMD. The Children’s Depression Rating Scale-Revised (CDRS-R), a widely used pediatric tool in pediatric depression trials, is not used because the scale is based on the HAMD and assesses similar content to the HAMD. A large portion of the CDRS-R items relate to school performance and friend socialization, which are difficult measures to adapt to hospitalized adolescents.

Other secondary efficacy outcomes include clinical response and remission at each time point up to the end of 4-week follow-up (Day 33). Anti-suicidal response is defined as ≥50% improvement in C-SSRS Ideation score compared to baseline and remission was defined as C-SSRS Ideation score = 0). Antidepressant response is defined as improvement ≥50% reduction in MADRS total score compared to baseline and remission was defined as a MADRS total score ≤ 12.

The change in self-reported suicidal ideation from Day 0 to Day 6 is another secondary efficacy outcome. We will utilize the first five items of the SSI (SSI-5) [26], which measure the hope to live, hope to die, reasons for living or dying, desire to actively attempt suicide, and passive suicidal thoughts. This scale will be self-assessed by the patient throughout the course of the study. Anxiety symptoms will be assessed by the 10 and 11 items of 17-item of the Hamilton Depression Inventory (HAMD-17) [27].

The evaluator conducted an interrater reliability exercise for all above scale (except for patient self-assessment scales) and found an intraclass correlation coefficient > 0.9.

### Safety assessments

General adverse events will be recorded using the Systematic Assessment for Treatment Emergent Events (SATEE) [28]. Other safety assessments include: (1) five items of the Brief Psychiatric Rating Scale (BPRS-5, hallucinations, grandiosity, suspiciousness, unusual thought content, conceptual disorganization); (2) the first item (elevated mood) of the Young Mania Rating Scale (YMRS) [29]; (3) Clinician Administered Dissociative Symptoms Scale (CADSS) [30], will be measured at baseline, then at 30 minutes and 24 hours after each infusion (Days 1, 2, 3 4, 5, 6, 12) and again at Days 12, 19 and 33. All these safety measures will be assessed covering the period since the previous assessment. Suicidal behavior will be assessed via the C-SSRS Behavior.

Cognitive function was assessed using the MATRICS consensus cognitive battery (MCCB) [31], administrated at baseline, Days 6 and 12. Four dimensions were selected from the MCCB, including processing speed, working memory, visual learning and verbal learning. Processing speed was measured by Brief Assessment of Cognition in Schizophrenia (BACS), Trail Making A test, and the Category Fluency test. Working memory was evaluated using the subtests Spatial Span of the Wechsler Memory Scale-III. Visual learning and verbal learning were assessed by the Hopkins Verbal Learning Test-Revised, and the Brief Visuospatial Memory Test-Revised, respectively. There are two test versions, version A and B, in visual learning and verbal learning, which had different topics but equal difficulty. In order to reduce potential learning effect in subjects, we used version A at baseline and Day 12, and version B at Day 6. The score of each dimension in MCCB was standardized to a T score with a mean of 50 and a standard deviation of 10, with higher score indicating better cognition.

During the follow-up phase, the probable dependence of the study drug will be assessed with the severity dependence scale (SDS) [32]. One item: “Did you wish you could stop?” is excluded from the present study because the study drug will be stopped during the follow-up phase. Thus only four items will be assessed. Each item is scored from 0 to 3. A cutoff point of 2/3 will be chosen to identify probable ketamine dependence. In addition, the visual analog scale (VAS [33], range 0–10) will be used to measure the aspects of craving for the study drug. The SDS and VAS will be assessed at Days 12, 19 and 33, covering the period since the previous assessment.

### Blood sample collection

Peripheral blood samples will be collected at baseline, Days 6 and 12 and processed into plasma, which will be used for testing BDNF, inflammatory factors, kynurenine metabolites, proteomics, metabolomics and other biological indicators. Whole blood will also be collected in Tempus tubes for direct isolation of RNA.

### Brain magnetic resonance imaging

Magnetic Resonance Imaging (MRI) scans will be performed at baseline, Days 6 and 12. All participants will be scanned Structural and functional MRI data on a 3.0 Tesla MRI system (Achieva X-series, Philips Medical Systems, Best, Netherlands). Foam pads and headphones will be used to minimize head motion and scanner noise. High-resolution T1-weighted images will be collected with a sagittal T1-weighted 3D turbo field echo (T1W 3D TFE) sequence (field of view 256 × 256 mm^2^; repetition time 8.2 ms; echo time 3.8 ms; view matrix 256 × 256; slice thickness 1 mm).

During the follow-up phase, the aforementioned efficacy and safety evaluations will be required to be completed even if the participants receive only one or two infusions.

**Fig. 2:**
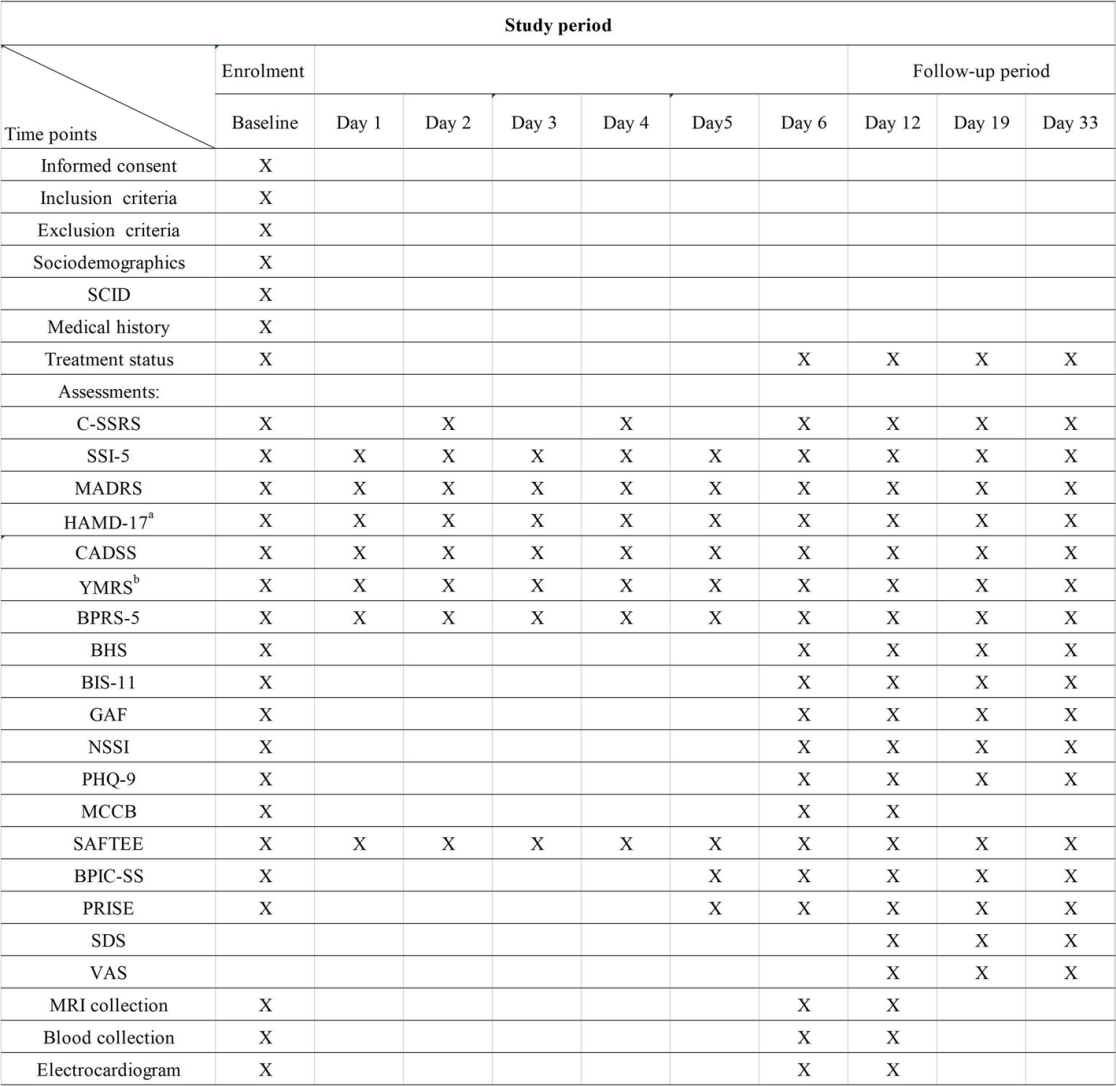
Measurements and time points. Abbreviations: SCID-5: Structured Clinical Interview for DSM-V; C-SSRS: Columbia suicide severity rating scale; MADRS: Montgomery–Asberg Depression Rating Scale; HAMD-17: 17-item Hamilton Rating Scale for Depression: SSI-5: The first five items of Baker Suicide Scale: HAMA: Hamilton Anxiety Scale [34]; CADSS: Clinician Administered Dissociative Symptoms Scale; YMRS: Young Mania Rating Scale; BPRS: Brief Psychiatric Rating Scale positive symptoms [35]; SDS: Severity Dependence Scale; VAS: Visual Analog Scale; MCCB: Measurement and Treatment Research to Improve Cognition in Schizophrenia Consensus Cognitive Battery; GAF: Global Assessment Function [36]; PHQ-9: Patien Health Questionnare-9 [37]; BPIC-SS: Bladder Pain/Interstitial Cystitis- Symptom Score [38]; SATEE: Systematic Assessment for Treatment Emergent Events; NSSI: Non-suicide Self-inflicted Injury

All measures and time points are shown in Fig. 2.

### Data management

All participant demographic and scale related results will be recorded in the case report form (CRF) and then stored in epidata (double data entry). To protect patient privacy, personal information such as names, contact numbers, and addresses that are not relevant to the data analysis will be removed from the data entry process. Numbering will be used to differentiate between patients. Also, the original CRFs are stored in a secure, convenient location throughout the study. Raw CRFs and epidata data are only accessible to the project leader and principal investigator.

Our study does not have the oversight of a data monitoring committee, but the subject group has a dedicated person to oversee.

## Statistical analysis

### Sample size calculation

Our study will be divided into two treatment cohorts, one for adolescents and the other for adults, and the two cohorts will be conducted independently, having separate randomization numbers. Power analysis determines sample size using a meta-analytic estimate of effect size = 0.85 for the change in clinician-rated suicidal ideation score for a single dose of ketamine vs control. This effect size requires a sample size of 22 participants per treatment group to achieve 80% power at two-sided significance level of 0.05. Assuming a 10% dropout rate after the first infusion, at least a sample size of 25 participants was needed for each treatment group. That is, at least 50 participants in adolescent cohort and 50 participants in adult cohort.

### Data analyses

Efficacy Data will be analyzed based on analysis sets that includes all randomized patients who complete both a baseline and Day 2 assessment with the C-SSRS. Safety analyses will be performed based on a safety analysis set that included all randomized participants who receive at least one infusion of study drug. Two treatment cohorts (adolescents and adults) will be independently statistically analyzed. Analysis will be performed using IBM SPSS Statistics version 27. The significance level for all statistical analyses is set at 0. 05.

### Baseline analyses

Baseline characteristics will be compared in groups using two-sample t-tests, chi-square tests, or Mann-Whitney U tests.

### Efficacy analyses

Linear mixed models will be used for efficacy analyses to assess changes from baseline to day 6 in C-SSRS Ideation and Intensity score, MADRS score and SSI-5 score, with time (Days 0, 2, 3 and 6), drug (esketamine and midazolam) and time-by-drug interactions as fixed factors, and total antidepressant class (selective serotonin reuptake inhibitor [SSRI] or non-SSRI), plus augmentation therapy, and respective baseline scores for the instruments as covariates. The Bonferroni-corrected simple effects post hoc tests will be used to examine differences in medications synchronized with significant interactions. Effect sizes (Cohen’s d) will be calculated using the mean difference between the two treatment groups at each time point. At the 6-day endpoint, a linear mixed models will be calculated based on intention-to-treat (ITT). Post hoc summaries of the proportion of participants who achieved clinical responses and remission will be provided, and differences between groups at each time point will be compared using two sample proportion tests.

To investigate the relationship between antidepressant effect and antisuicidal effect, we will use correlation analyses to explore the correlation between reduction in MADRS score and reduction in C-SSRS Ideation or Intensity scorein each group; Mediation analysis will be used to examine whether the effect of the intervention on overall depressive symptoms mediates the effect of the intervention on suicidal ideation. Mediated analyses will be conducted using the process v3.5 in SPSS. The model will use the study drug as the independent variable, the reduction in C-SSRS Ideation or Intensity score as the dependent variable, and the decrease in MADRS scores as mediator.

### Safety analysis

The frequency distribution of treatment-emergent adverse events and suicidal behaviors will be depicted, while CADSS, BPRS-5 and YMRS item 1 scores will be compared between groups at each time point using univariate tests. Differences in SDS and VAS scores between groups will be analyzed by two independent samples t-tests. The proportion of participants meeting study drug dependence criteria at each time point will be compared between groups using a two-sample proportion test. A mixed-effects repeated measures model was conducted to examined differences in four dimensions of MCCB between groups from baseline to endpoint (Day 12).

## Discussion

This manuscript describes the study protocol of a randomized double-blind controlled trial of three infusions of intravenous esketamine in MDD patients with suicidal ideation, with the primary objective of exploring whether esketamine treatment reduces suicidal ideation as well as depressive symptom in this population.

Suicidal ideation in MDD patients is a serious and urgent condition that requires immediate treatment. However, acute pharmacological management of suicidal risk in MDD remains relatively unstudied in clinical practice. Antidepressants have been shown in numerous studies to reduce overall suicide risk, but they are slow to take effect, while some studies have found an increased risk of suicide within the first month after SSRI treatment. In the United States, the issue is followed by the FDA black box warning on the use of SSRIs in children, adolescents and young adults [39]. In addition, MDD patients with suicidal ideation is indicative of ECT [40]. One study has shown that ECT has better antisuicidal effects in patients with monophasic disorder and bipolar depression compared with psychopharmacological treatment [41]. However, there are significant drawbacks to the use of ECT, and public acceptance is relatively low (10–32%) [42]. Repeated anesthesia carries the potential for serious cognitive risk and can be very distressing for those with memory impairment after ECT [43]. Ketamine appears to show a more favorable cognitive side effect profile than ECT, and there is evidence that ketamine has similar and faster antidepressant efficacy than ECT, despite the different sources of patient recruitment [44]. The conclusions drawn from our study are expected to provide an evidence base for treatment options for patients with concomitant suicidal depression.

In terms of study population, our study will recruit not only adults, but also adolescents. Previous ketamine-related trials have mostly focused on adults but lack evidences from adolescents. Only one open-label trials and one randomized controlled trial have reported antidepressant efficacy of ketamine in adolescents [45, 46]. In addition, two adolescent case reports assessing measures of suicidal ideation both found ketamine has rapid antisuicidal effects [47, 48]. These findings lack further randomized double-blind controlled studies. In addition, one study has reported a high level of acceptance of parental attitudes toward ketamine use in adolescent mood disorders with suicide ideation [49]. This implies the feasibility of replicating our study in adolescents with suicidal ideation.

In terms of intervention modality, we chose three-dose of intravenous esketamine. Although data from chronic pain management suggest that oral ketamine can often be used safely for longer periods of time [50], there are limited reports of antidepressant studies of oral esketamine, with only a few small studies reporting the antidepressant properties of oral ketamine [51–54]. Bioavailability considerations are a major concern. The bioavailability of oral ketamine is variable and low compared to intravenous ketamine, and the absorption rate of oral ketamine appears to vary considerably between and within patients [55, 56]. Some oral (es) ketamine studies have shown antidepressant effects within a few hours of administration, but most studies have shown such effects only after several weeks of treatment [54]. In addition, a systematic review and meta-analysis showed that intravenous ketamine showed more significant overall response and remission rates, as well as lower withdrawal rates due to adverse events, compared to intranasal esketamine [57]. Previous studies did show a prolonged duration of response after repeated dosing compared to single dose ketamine infusion [7–9]. MDD patients with suicidal ideation need rapid relief of suicidal ideation, and from the above, repeated intravenous dosing seems to be the preferred dosing method.

A dose of 0.5 mg/kg ketamine is the most commonly used dose in depression trials, and esketamine is considered to be 2 times more effective than racemic ketamine as an anesthetic [58–60]. Therefore, we selected 0.25 mg/kg esketamine for the intervention. Studies have shown that there may be a dose-response relationship between the effects of ketamine on depressive symptoms with TRD. Previous studies found that the use of 0.5 mg/kg ketamine was more effective than lower doses of ketamine in adult MDD patients, and increasing the dose from 0.5 to 0.75 mg/kg may effective in some patients [61, 62]. Higher doses of ketamine may result in more pronounced acute side effects [60]. In addition, ketamine is an addictive drug, and it is unclear whether its use as a treatment for depression may lead to increased rates of abuse. Bladder toxicity is another potentially worrisome side effect that can result from chronic ketamine exposure [63]. In the future direction, not only further controlled trials with more flexible doses are needed, but also an in-depth exploration of the mechanism of action of ketamine.

## Conclusion

The results of this randomized controlled trial are expected to have a potentially important impact on the treatment of MDD patients with suicidal ideation. Our data may support the use of repeated esketamine infusions, which could meet the urgent need for rapid relief of suicidal ideation as well as depressive symptoms in this population. We will begin formal recruitment in January 1, 2021 and recruitment will continue through December 31, 2023.

## Data Availability

Not applicable.

## Abbreviations

MDD: Major depressive disorder
RCT: randomized, double-blind, placebo-controlled trial
NMDA: N-methyl-D-aspartate
FDA: Food and Drug Administration
TRD: refractory depression
ECT: electroconvulsive therapy
CRF: the case report form
HDEI: the Hospital Department of Education and Information
rTMS: repetitive transcranial magnetic stimulation
SpO2: Blood oxygen saturation
BACS: Brief Assessment of Cognition in Schizophrenia
MRI: Magnetic Resonance Imaging
SSRI: selective serotonin reuptake inhibitor
ITT: intention-to-treat
SCID-5: Structured Clinical Interview for DSM-V
C-SSRS: Columbia suicide severity rating scale
MADRS: Montgomery–Asberg Depression Rating Scale
HAMD-17: 17-item Hamilton Rating Scale for Depression
SSI-5: The first five items of Baker Suicide Scale
HAMA: Hamilton Anxiety Scale
CADSS: Clinician Administered Dissociative Symptoms Scale
YMRS: Young Mania Rating Scale
BPRS: Brief Psychiatric Rating Scale positive symptoms
SDS: Severity Dependence Scale
VAS: Visual Analog Scale
MCCB: Measurement and Treatment Research to Improve Cognition in Schizophrenia Consensus Cognitive Battery
GAF: Global Assessment Function
PHQ-9: Patien Health Questionnare-9
BPIC-SS: Bladder Pain/Interstitial Cystitis- Symptom Score
SATEE: Systematic Assessment for Treatment Emergent Events
NSSI: Non-suicide Self-inflicted Injury
